# Disruptions and Adaptations in End-of-Life Care Delivery for Homebound Patients During COVID-19

**DOI:** 10.1093/geroni/igab046.2056

**Published:** 2021-12-17

**Authors:** Emily Franzosa, Patricia Kim, Meng Zhang, Jennifer Reckrey, Sara Lubetsky, Emily Xu, Katherine Ornstein

**Affiliations:** 1 Icahn School of Medicine at Mount Sinai, Icahn School of Medicine at Mount Sinai, New York, United States; 2 Icahn School of Medicine at Mount Sinai, New York, New York, United States; 3 Icahn School of medicine at Mount Sinai, New York, New York, United States

## Abstract

The initial COVID-19 pandemic surge in New York City caused widespread delays and disruption in end-of-life services. This study examined the impact of disruptions among homebound adults in an HBPC practice who died between March-June 2020. Through an in-depth mixed-methods chart review, we identified 113 patient deaths (mean age: 87, 73% female, 67% with dementia). Forty-nine (43%) of deaths occurred in April 2020. Through a content analysis of clinician notes, we identified key COVID-related themes, including a shift to intensive phone-based care and to a lesser degree, telehealth; delays in hospice referrals and admissions; and an increase in treatment for behavioral symptoms. Our analysis also demonstrated the central role of family and paid caregivers in coordinating care, and efforts by patients, caregivers and providers to avoid hospital admissions. These findings demonstrate the importance of care coordination across medical, home and community partners to support end-of-life care in emergencies and beyond.

